# A double-blind placebo-controlled cross-over clinical trial of DONepezil In Posterior cortical atrophy due to underlying Alzheimer's Disease: DONIPAD study

**DOI:** 10.1186/s13195-018-0363-1

**Published:** 2018-05-01

**Authors:** Basil H. Ridha, Sebastian Crutch, Dawn Cutler, Christopher Frost, William Knight, Suzie Barker, Norah Epie, Elizabeth K. Warrington, Riitta Kukkastenvehmas, Jane Douglas, Martin N. Rossor

**Affiliations:** 1 Dementia Research Centre, UCL Institute of Neurology, Queen Square, London, WC1N 3BG UK; 2 London School of Hygiene and Tropical Medicine, University of London, London, UK

**Keywords:** Posterior cortical atrophy, Alzheimer’s disease, Donepezil, Clinical trial

## Abstract

**Background:**

The study investigated whether donepezil exerts symptomatic benefit in patients with posterior cortical atrophy (PCA), an atypical variant of Alzheimer’s disease.

**Methods:**

A single-centre, double-blind, placebo-controlled, cross-over clinical trial was performed to assess the efficacy of donepezil in patients with PCA. Each patient received either donepezil (5 mg once daily in the first 6 weeks and 10 mg once daily in the second 6 weeks) or placebo for 12 weeks. After a 2-week washout period, each patient received the other treatment arm during the following 12 weeks followed by another 2-week washout period. The primary outcome was the Mini-Mental State Examination (MMSE) at 12 weeks. Secondary outcome measures were five neuropsychological tests reflecting parieto-occipital function. Intention-to-treat analysis was used. For each outcome measure, carry-over effects were first assessed. If present, then analysis was restricted to the first 12-week period. Otherwise, the standard approach to the analysis of a 2 × 2 cross-over trial was used.

**Results:**

Eighteen patients (13 females) were recruited (mean age 61.6 years). There was a protocol violation in one patient, who subsequently withdrew from the study due to gastrointestinal side effects. There was statistically significant (*p* < 0.05) evidence of a carry-over effect on MMSE. Therefore, the analysis of treatment effect on MMSE was restricted to the first 12-week period. Treatment effect at 6 weeks was statistically significant (difference = 2.5 in favour of donepezil, 95% CI 0.1 to 5.0, *p* < 0.05). Treatment effect at 12 weeks was close, but not statistically significant (difference = 2.0 in favour of donepezil, 95% CI –0.1 to 4.5, *p* > 0.05). There were no statistically significant treatment effects on any of the five neuropsychological tests, except for digit span at 12 weeks (higher by 0.5 digits in favour of placebo, 95% CI 0.1 to 0.9). Gastrointestinal side effects occurred most frequently, affecting 13/18 subjects (72%), and were the cause of study discontinuation in one subject. Nightmares and vivid dreams occurred in 8/18 subjects (44%), and were statistically more frequent during treatment with donepezil.

**Conclusions:**

In this small study, there was no statistically significant treatment effect of donepezil on the primary outcome measure (MMSE score at 12 weeks) in PCA patients, who appear to be particularly susceptible to the development of nightmares and vivid dreams when treated.

**Trial registration:**

Trial registration: Current Controlled Trials ISRCTN22636071. Retrospectively registered 19 May 2010

## Background

Alzheimer’s disease (AD) typically presents with progressive impairment of episodic memory as a result of degeneration of medial temporal lobe structures before the involvement of other cortical regions causing widespread global cognitive impairment. Cholinesterase inhibitors (AChEI) have been shown to provide symptomatic benefit in patients with mild to moderate AD [1]. A small but significant proportion of patients with AD-type pathology present with visual perceptual or visual spatial dysfunction, apraxia, dyscalculia or alexia reflecting posterior cortical dysfunction, with relative preservation of episodic memory. Structural brain imaging in these patients reveals parieto-occipital atrophy (posterior cortical atrophy (PCA)) [2]. Only one case report suggested potential therapeutic benefit from taking AChEI in a patient with PCA [3]. Otherwise, there have been no studies assessing the effectiveness of AChEI specifically in patients with PCA due to underlying AD.

## Methods

### Study aim

This study assessed the efficacy of donepezil, a licensed AChEI, in patients with PCA due to underlying AD.

### Study design

This is a single-centre, double-blind, placebo-controlled, cross-over clinical trial assessing the efficacy of donepezil in patients with PCA presumed to be due to underlying AD. The study was approved by the Local Ethics Committee and was subject to inspection by the Medicines and Healthcare Products Regulatory Agency in the UK. The study was conducted during the period April 2003–December 2011. Both patients and clinical investigators remained blinded to study treatment for the whole duration of the study, even after the statistical analysis of the whole study was completed.

**Fig. 1:**
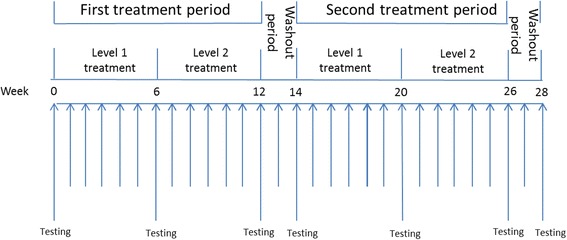
DONIPAD study timeline. DONIPAD DONepezil In Posterior cortical atrophy due to underlying Alzheimer’s Disease

The study timeline is shown in Fig. 1. During the first treatment period (weeks 1–12), patients were randomised to receive either donepezil or placebo. During the first 6 weeks of the first treatment period (weeks 1–6), patients received either donepezil 5 mg once daily or placebo once daily (level-one treatment). During the following 6 weeks of the first treatment period (weeks 7–12), the dose of donepezil was increased to 10 mg once daily in the active treatment arm or patients were continued on placebo in the placebo arm (level-two treatment). The first treatment period was followed by a 2-week washout period (weeks 13–14). During the second treatment period (weeks 15–26) the treatment arms were crossed over using a similar dose escalation as in the first treatment period: that is, level-one treatment during the first 6 weeks (weeks 15–20) and level-two treatment during the following 6 weeks (weeks 21–26). The second treatment period was followed by a second 2-week washout period (weeks 27–28).

### Study participants

Patients with a clinical diagnosis of PCA due to underlying AD were recruited from the Cognitive Disorders Clinic at the National Hospital for Neurology and Neurosurgery, London, UK. Patients with clinical features or work-up suggestive of an underlying pathology other than AD (dementia with Lewy bodies, corticobasal degeneration, prion disease) were excluded (e.g. visual hallucinations, reduplicative phenomena, parkinsonism, alien limb syndrome, asymmetric dystonia and myoclonus, ataxia). All patients signed consent forms with assent from the next of kin. At the time of study design, there were no published clinical criteria for the diagnosis of PCA. Instead, all patients met the inclusion and exclusion criteria presented in Table 1. Retrospectively, all patients met the Mendez et al. and Tang-Wai et al. criteria for PCA [4, 5].

**Table 1:** Inclusion and exclusion criteria

Inclusion criteria
1. Clinical diagnosis of PCA based on clinical presentation and formal neuropsychological assessment suggestive of significant visuo-perceptual or visuo-spatial disturbance, visual disorientation, apraxia, dysgraphia or dyscalculia in the context of relatively preserved memory 2. Performance below the 5th percentile on two of the following four tests of parietal lobe function: • Jackson and Warrington calculation test [21] • number location visual spatial test [9] • object decision visual perceptual test [9] • Baxter and Warrington spelling test [22] 3. Verbal memory test score above the 5th percentile on a neuropsychological assessment such as the verbal recognition memory test [23] 4. Absence of alternative cause of cognitive impairment based on clinical history and examination, and available investigations including blood tests, electroencephalograph, MRI of brain and formal neuropsychological assessment
Exclusion criteria
1. Significant neurological or psychiatric disease that may affect cognition other than Alzheimer’s disease 2. Significant systemic disease that may deteriorate or affect the patient’s safety or the ability to cooperate with the study protocol 3. Medications with the potential to affect cognition unless maintained on a stable dose for at least 3 months prior to week 0 visit 4. Patients with lactose intolerance 5. Known hypersensitivity to donepezil hydrochloride, piperidine derivatives, or to any excipients used in the formulations

*MRI* magnetic resonance imaging, *PCA* posterior cortical atrophy

### Outcome measures

The primary end point was change in the Mini-Mental State Examination (MMSE) score at 12 weeks [6]. The MMSE has been used as a cognitive outcome measure in studies assessing the efficacy of cholinesterase inhibitors in AD [7]. The MMSE has also been used clinically to assess eligibility for treatment with a cholinesterase inhibitor [8]. The MMSE is relatively short to administer and so can be conveniently administered repeatedly over a 28-week study period. We used the same three words for testing word repetition and verbal recall throughout. Although this increases the risk of learning effects on repeated testing, it maintains consistency in the level of test difficulty throughout the study. Secondary end points were five tests reflecting parieto-occipital function: number location test [9]; simple calculation test (unpublished data); letter cancellation test [10]; dot counting test [9]; and digit span [11]. The tests were performed at baseline (week 0) and at the end of weeks 6, 12, 14, 20, 26 and 28 (Fig. 1). Adverse events were recorded throughout the study to monitor safety.

### Statistical analysis

Analysis of each outcome variable was pre-specified and followed the principles described by Jones and Kenward [12] for the analysis of 2 × 2 cross-over trials with a baseline measurement in each period. Since a number of the outcome variables have floor and ceiling effects and are not normally distributed, bias-corrected and accelerated bootstrap confidence intervals (CIs) calculated from 2000 bootstrap samples were used throughout and used to assess whether results were statistically significant.

For each variable, two tests for carry-over were performed prior to assessing treatment efficacy. First, a test for carry-over was carried out using the baseline values immediately prior to each period alone. Second, a standard test of a direct treatment-by-period interaction (which can also be interpreted as a test for carry-over) was carried out using the changes from baseline to 12 weeks.

If either of the tests for carry-over was statistically significant (*p* < 0.05, two-sided, assessed from the bootstrap CI), then tests of treatment efficacy were carried out by comparing changes from baseline in the first period alone. The primary analysis was a *t* test (with bootstrap CI) comparing changes from baseline to 12 weeks in the donepezil and placebo groups. A secondary analysis compared analogous changes from baseline to 6 weeks.

If neither of the tests for carry-over yielded statistically significant results, then the treatment effect was estimated ignoring the baseline measures and using the standard approach to the analysis of a 2 × 2 cross-over trial. This standard approach used a repeated-measures two-way analysis of variance model (with bootstrap CI) with period and treatment as the predictor variables. This permits a within-subject, period-adjusted treatment effect to be estimated. The primary analysis used the 12-week data alone and a secondary analysis used the 6-week data alone.

## Results

### Participant characteristics

Eighteen patients were screened and met the inclusion and exclusion criteria. The mean age was 61.6 years (range 50–74), and the male:female ratio was 5:13. Table 2 presents baseline characteristics according to the randomisation group.

**Table 2:** Baseline characteristics of randomised groups

	Placebo then donepezil (*n* = 10)	Donepezil then placebo (*n* = 8)
Gender	4 male, 6 female	1 male, 7 female
Handedness	10 right	3 left, 5 right
Age (years)	60.2 (7.3)	62.9 (6.0)
Disease duration (years)	3.3 (1.9)^a^	3.7 (1.5)
MMSE	23.9 (4.0)	22.6 (3.2)
Simple calculation	6.9 (1.4)^b^	7.3 (1.0)^a^
Number location	3.9 (2.8)	3.8 (2.9)
Letter cancellation	59.8 (21.5)	91.9 (19.7)
Dot counting	5.3 (2.6)^a^	5.1 (3.6)^a^
Digit span	13.4 (3.0)^a^	13.6 (4.4)^a^

Data presented as mean (standard deviation) unless otherwise stated

*MMSE* Mini-Mental State Examination

^a^One missing value

^b^Two missing values

AD pathology was confirmed at post-mortem in two patients who completed the study and donated their brain to the Queen Square Brain Bank for Neurological Disorders, UCL Institute of Neurology, London, UK. Lumbar puncture done during clinical diagnostic work-up on one of the patients revealed normal total CSF Tau concentration at 566 ng/ml and low CSF Aβ_1–42_ concentration at 206 ng/ml, supportive of the presence of AD pathology.

### Protocol violation and participant withdrawal

The protocol was not followed correctly for one female participant aged 58 years. Instead of increasing the dose from level-one treatment to level-two treatment during the first treatment period at the end of week 6, the patient was erroneously given level-one treatment of the cross-over second treatment period. The error was discovered at the week 14 visit. A decision was taken to maintain her on level-two treatment of the second treatment period over the following 6 weeks. The intention was to repeat level-one and level-two treatments of the first treatment period of the study after a 2-week washout period. However, the patient decided to withdraw from the trial due to the development of gastrointestinal side effects soon after repeating level-one treatment of the first treatment period.

We present the results analysed according to the principles of intention to treat (ITT) with the patient’s data included up to the point at which she withdrew and analysed according to the treatment that she should have received at each visit. We also performed per-protocol analysis with this patient’s data omitted from the point at which the wrong treatment was given. As there was no significant difference between the two analysis methods, only the ITT analysis will be presented.

### Mini-Mental State Examination

**Fig. 2:**
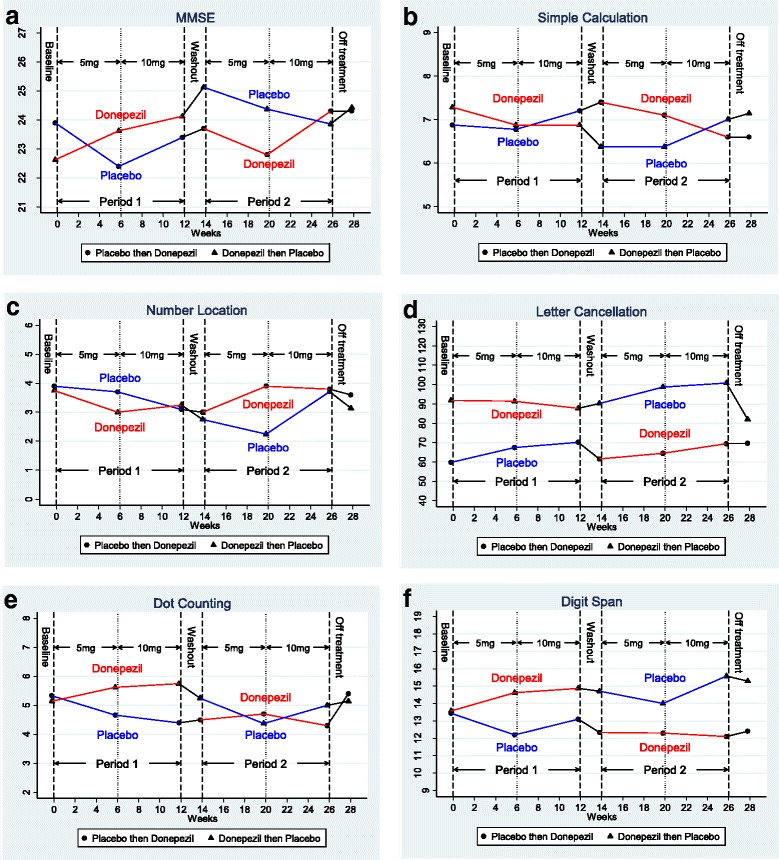
Mean value of outcome measures at different time points: (**a**) MMSE, (**b**) simple calculation test, (**c**) number location test, (**d**) letter cancellation test, (**e**) dot counting, (**f**) digit span. MMSE Mini-Mental State Examination

**Fig. 3:**
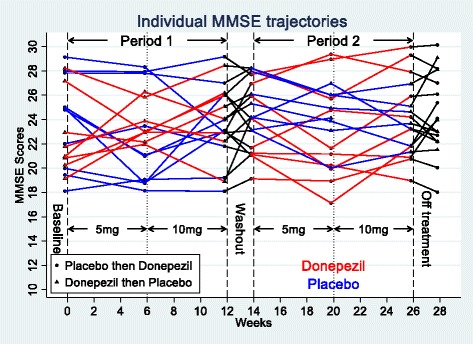
Individual MMSE trajectories. MMSE Mini-Mental State Examination

There was statistically significant (*p* < 0.05) evidence of carry-over from the comparison of values immediately prior to each treatment period (Table 3). Mean baseline MMSE score of patients randomised to placebo in the first treatment period was 23.9, and at the end of the washout period was 23.7. Mean baseline MMSE score of patients randomised to donepezil in the first treatment period was 22.6, and at the end of the washout prior to starting placebo was 25.1 (Figs. 2a and 3).

**Table 3:** Summary of data analysis of changes in MMSE and five neuropsychometric tests reflecting parieto-occipital function

	Change from baseline of first treatment period to baseline visit of second treatment period (test of carry-over from baseline values)			First treatment period: change from baseline visit to week 6 visit			First treatment period: change from baseline visit to week 12 visit		
	Donepezil (*n* = 8), mean (SD)	Placebo (*n* = 10), mean (SD)	Difference (95% CI)	Donepezil (*n* = 8), mean (SD)	Placebo (*n* = 10), mean (SD)	Difference (95% CI)	Donepezil (*n* = 8), mean (SD)	Placebo (*n* = 10), mean (SD)	Difference (95% CI)
MMSE	2.5 (1.4)	−0.2 (1.7)	2.7 (1.3, 4.0)	1.0 (3.1)	−1.5 (2.4)	2.5 (0.1, 5.0)	1.5 (2.7)	−0.5 (2.4)	2.0 (−0.1, 4.5)
Simple calculation	−0.9 (0.7)^a^	0.6 (1.1)^b^	−1.5 (−2.6, –0.8)^c^	−0.1 (0.7)^a^	− 0.1 (0.8)^b^	–0.0 (−0.7, 0.8)^c^	0.0 (1.3)^a^	0.4 (1.1)^b^	−0.4 (−1.4, 0.8)^c^
				Donepezil vs placebo 6-week comparison			Donepezil vs placebo 12-week comparison		
				Donepezil first (*n* = 8), mean (SD)	Placebo first (*n* = 10), mean (SD)	Combined (*n* = 18), mean (95% CI)	Donepezil first (*n* = 8), mean (SD)	Placebo first (*n* = 10), mean (SD)	Combined (*n* = 18), mean (95% CI)
Number location	−1.0 (2.2)	−0.9 (1.3)	−0.1 (−1.7, 1.5)	0.8 (1.8)	0.2 (1.5)	0.5 (− 0.1, 1.5)	0.0 (2.4)^a^	0.7 (2.4)	0.4 (−0.8, 1.4)^a^
Letter cancellation	−0 (25)^a^	2 (21)	−2 (−22, 19)^a^	−7 (39)	−3 (15)	−5 (−19, 8)	−14 (35)^a^	−1 (14)	−8 (−23, 4)^a^
Dot counting	−0.3 (1.5)^a^	−1.0 (2.4)^a^	0.7 (−1.4, 2.3)^b^	1.3 (1.8)	−0.2 (1.3)^a^	0.5 (−0.2, 1.2)^a^	1.3 (0.8)^a^	−0.1 (1.7)	0.6 (−0.1, 1.1)^a^
Digit span	0.3 (1.5)^b^	0.0 (1.3)^b^	0.3 (−0.9, 2.0)^d^	0.6 (1.9)	0.1 (1.6)	0.4 (−0.4, 1.2)	0.0 (1.0)^a^	−1 (0.8)	−0.5 (−0.9, −0.1)^a^

*CI* confidence interval, *MMSE* Mini-Mental State Examination, *SD* standard deviation

^a^One missing value

^b^Two missing values

^c^Three missing values

^d^Four missing values

Because of this evidence of carry-over, efficacy was assessed by comparing changes in the first treatment period only. The treatment effect at 12 weeks was not statistically significant but close (2.0 in favour of donepezil, 95% CI –0.1 to 4.5), whilst that at 6 weeks was just statistically significant (2.5 in favour of donepezil, 95% CI 0.1 to 5.0) (Table 3, Fig. 2a).

### Simple calculation test

There was statistically significant evidence of carry-over from the comparison of values immediately prior to each treatment period (Table 3). Therefore, treatment effects were assessed by comparing changes in the first treatment period only. Treatment effects at 6 and 12 weeks were not statistically significant (Table 3, Fig. 2b).

### Number location spatial test

Tests for carry-over were not statistically significant (Table 3). Therefore, treatment effects were estimated using the standard approach to the analysis of a cross-over trial. There was no statistically significant difference at 6 or 12 weeks of treatment (Table 3, Fig. 2c).

### Letter cancellation test

Tests for carry-over were not statistically significant (Table 3). Therefore, treatment effects were estimated in the standard approach to the analysis of a cross-over trial. There was no statistically significant difference at 6 or 12 weeks of treatment (Table 3, Fig. 2d).

### Dot counting test

Tests for carry-over were not statistically significant (Table 3). Therefore, treatment effects were estimated in the standard approach to the analysis of a cross-over trial. There was no statistically significant difference at 6 or 12 weeks of treatment (Table 3, Fig. 2e).

### Digit span

Tests for carry-over were not statistically significant (Table 3). Therefore, treatment effects were estimated in the standard approach to the analysis of a cross-over trial. There was no statistically significant difference at 6 weeks of treatment. After adjusting for period effects, the mean digit span was 0.5 digits higher at 12 weeks when patients were receiving placebo (Table 3, Fig. 2f).

### Safety results

There were no serious adverse reactions reported. One patient discontinued the study due to abdominal cramps and nausea.

The following side effects were reported and are presented in order of frequency:

1. Gastrointestinal side effects (nausea, abdominal cramps, diarrhoea) occurred in 13 subjects (72%): seven (39%) only whilst on donepezil, four (22%) only whilst on placebo and two (11%) during both treatment periods. One of these two subjects was the one in whom the protocol was not followed correctly. The subject only reported gastrointestinal side effects whilst on donepezil, and ultimately discontinued because of these.
2. Nightmares and vivid dreams occurred in eight (44%) subjects: seven (39%) only whilst on donepezil, one (6%) during both treatment periods and none only whilst on placebo.
3. Anxiety/low mood occurred in six (33%) subjects: three (17%) only whilst on donepezil, one (6%) only whilst on placebo and two (11%) during both treatment periods.
4. Headache occurred in five (28%) subjects: one (6%) only whilst on donepezil, two (11%) only whilst on placebo and two (11%) during both treatment periods.
5. Dizziness occurred in three (17%) subjects: one (6%) whilst on donepezil, one (6%) only whilst on placebo and one (1%) during both treatment periods.
6. Muscular aches, pains and/or jerking movements occurred in three (17%) subjects: two (11%) only whilst on donepezil and one (6%) only whilst on placebo.

Out of these, the only statistically significant side effect was nightmares and vivid dreams, which occurred more frequently during donepezil than placebo treatment periods (*p* = 0.02, McNemar’s test, Fisher’s exact *p* value).

## Discussion

All patients screened met the inclusion and exclusion criteria. Patients with PCA due to underlying AD were already known to the clinical investigators as they were under their clinical care. This allowed rigorous pre-screening based on their clinical assessment, and so only patients who were highly likely to fulfil the study criteria were invited to participate.

The study was conducted over a long period (8.7 years). This reflected the relative rarity of this atypical variant of AD and the difficulties in identifying drug-naïve subjects who have not yet been started on a cholinesterase inhibitor.

As would be anticipated in a small trial, some baseline imbalances were seen, with the imbalance in baseline letter cancellation scores being the most marked. Since this is a cross-over trial, with each patient acting as their own control, these imbalances matter much less than would be the case in a parallel-group trial. In our analysis we could have additionally adjusted for the baseline values made immediately prior to each period, but did not do so, preferring to use the method we had pre-specified in our statistical analysis plan as recommended in the then current edition of Jones and Kenward [12].

In our primary analysis there was no statistically significant treatment effect of donepezil on the primary outcome measure: change in MMSE at 12 weeks. However, since this is a small cross-over trial, all of our results, both positive and negative, must be interpreted cautiously. Some of our results do provide evidence for a beneficial effect of donepezil on MMSE in patients with PCA due to underlying AD. Firstly, there was statistically significant evidence of a carry-over effect suggesting that the effect of donepezil may have continued after the 2-week washout period, with the largest effects only being seen after the washout period. Secondly, during the first treatment period, there was statistically significant difference in the change in the MMSE at 6 weeks (MMSE difference = 2.5, 95% CI 0.1 to 5.0, *p* < 0.05). We reiterate, however, that the small sample size, the borderline nature of the statistical results, and the fact that the direction of the baseline imbalance coupled with regression to the mean are likely to have exaggerated the differences and mean that the results should be interpreted cautiously.

There was no statistically significant improvement in any of the neuropsychological tests relating to parieto-occipital function as a result of treatment with donepezil in patients with PCA due to underlying AD. In fact, there was statistically significant evidence for a slight reduction of digit span by 0.5 digits (*p* < 0.05) after 12 weeks of treatment with donepezil. However, this result should be interpreted cautiously in view of the number of statistical tests carried out and the consequent increased risk of false positive findings.

The most commonly reported adverse events were gastrointestinal (nausea, abdominal cramps or diarrhoea), affecting 67% of patients. These are well-recognised side effects of donepezil. Safety data from one phase II trial and three phase III trials revealed that gastrointestinal side effects occurred in 44% of patients receiving 10 mg donepezil compared to 22% of patients receiving placebo [13].

A study by Farlow *et al.* [14] identified an increased incidence of adverse events in patients with weight below 55 kg compared with those with higher weights. Although our study used significantly lower daily doses of donepezil of 5 mg and 10 mg, it would have been helpful to have recorded subjects’ weights in case weight still influenced the risk of developing side effects at these lower doses.

Nightmares and vivid dreams have been reported to occur in AD patients who have been prescribed donepezil, particularly if taken close to bedtime, and resolved once discontinued or when dosing time was changed to the morning [15–17].

Of our patients with PCA due to underlying AD, 44% experienced nightmares or vivid dream whilst taking donepezil and none during the placebo period alone. Singer et al. [17] reported nightmares to occur in eight out of 103 (8%) AD patients. Previous clinical trials of donepezil in AD found that insomnia and other sleep-related symptoms occurred in 8–18% of patients receiving donepezil compared with 4–6% of patients receiving placebo [18–20]. The higher incidence of nightmare and vivid dreams in our study may indicate that patients with PCA due to underlying AD may be particularly susceptible for the development of nightmares and vivid dreams due to disproportionate parieto-occipital dysfunction in this patient group.

There are several limitations to this study. The sample size of the study is small. PCA is relatively rare so it was challenging to find patients fulfilling the specified inclusion/exclusion criteria at a single site. AD is not the only cause of a clinical presentation of PCA; therefore the diagnosis of AD as the underlying cause of PCA is at best probable. With one exception, participating patients did not have CSF Tau and Aβ_1–42_ measurement or amyloid PET imaging as in-vivo biomarkers for the presence of AD pathology. However, AD was pathologically confirmed in two of the participating patients who subsequently died. Other conditions that can present with PCA include dementia with Lewy bodies, corticobasal degeneration and prion disease. None of our patients exhibited any neurological features to suggest any of these. Although this was a double-blind trial, the frequent occurrence of predictable side effects such as gastrointestinal side effects may have caused patients and clinical investigators to sometimes guess what treatment they were on. The washout period may have been too short, as evidenced by the carry-over effect on the MMSE and simple calculation test. The outcome measures are relatively crude, variable and influenced by patient and rater factors. In addition, learning may have occurred due to repeated testing over a short study period. The improvement in the MMSE rather than specific tests of biparietal function may relate to subtle improvements in relatively spared cognitive domains such as episodic memory and orientation, which are the MMSE’s predominant focus.

## Conclusions

This small, double-blind, placebo-controlled, cross-over study found no statistically significant treatment effect on the MMSE at 12 weeks (primary outcome measure) in patients with PCA.

Known common gastrointestinal side effects of donepezil occurred in the course of the study. The frequency of nightmares and vivid dreams occurred significantly more commonly in association with donepezil treatment, and possibly more commonly than reported previously in patients with the typical amnestic presentation of AD. Patients with PCA may be particularly vulnerable to these side effects.

## Abbreviations

AChEI: Acetylcholinesterase inhibitor
AD: Alzheimer’s disease
CI: Confidence interval
DONIPAD: DONepezil In Posterior cortical atrophy due to underlying Alzheimer’s Disease
MMSE: Mini-Mental State Examination
PCA: Posterior cortical atrophy
